# Personality, Category, and Cross-Linguistic Speech Sound Processing: A Connectivistic View

**DOI:** 10.1155/2014/586504

**Published:** 2014-03-13

**Authors:** Yizhou Lan, Will X. Y. Li

**Affiliations:** ^1^Department of Chinese, Translation and Linguistics, City University of Hong Kong, Hong Kong; ^2^Department of Electronic Engineering, City University of Hong Kong, Hong Kong

## Abstract

Category formation of human perception is a vital part of cognitive ability. The disciplines of neuroscience and linguistics, however, seldom mention it in the marrying of the two. The present study reviews the neurological view of language acquisition as normalization of incoming speech signal, and attempts to suggest how speech sound category formation may connect personality with second language speech perception. Through a questionnaire, (being thick or thin) ego boundary, a correlate found to be related to category formation, was proven a positive indicator of personality types. Following the qualitative study, thick boundary and thin boundary English learners native in Cantonese were given a speech-signal perception test using an ABX discrimination task protocol. Results showed that thick-boundary learners performed significantly lower in accuracy rate than thin-boundary learners. It was implied that differences in personality do have an impact on language learning.

## 1. Introduction

The connection between personality and language acquisition has been under inquiry for many decades. In the study of both neuropsychology and linguistics, scholars have different views of how extravert and introvert personalities may have an influence on the rate of learning a first [1–3] or second [4–7] language. Some argue that introverts are better learners because of their reflective habits of learning, but more support extravert because of the rich incentives and resourcefulness. The debate was ongoing, but few had jumped out of a flaw that personality is a single and independent factor influencing language, but, in fact, other mediating variables such as input and linguistic experience also exist. Personality can only influence language, in a subtle but substantial process, through more fine-grained cognitive device of the human mind. The current study wishes to focus on one aspect of language acquisition, speech, and search for empirical evidence as well as theoretical implications of the following proposal: whether personality can be seen as a factor, through the effect of category formation, in influencing cross-linguistic speech perception accuracy.

Categorization is a human capacity to translate the chaotic signal in the phenomenal world into economic, clear-cut, and readily usable clusters of information which can be an integral part of cognitive capability. Without it, smooth reception and transmission of information would not be possible [8]. Language, a vital part of cognition, will lose its function for perception and production as well without categorization.

Having a different personality may result in different categorization procedures. Psychologists denote extravert and introvert persons in that their external and internal ego boundary is different. extravert persons were thick in their external and internal boundaries, whereas introvert ones were thin in them [9–14]. Individuals with thick external boundaries tend to have wide-scattered categories, whereas those with thin external boundaries may have larger category density. The boundary determines how the individual would respond to pervasive environmental stimuli [12].

For thick-boundary persons, their attentions were directed by motivation, goals, and self-consciousness, and thus pay more attention to salient cues on certain stimuli. According to Ehrman [15], Hartmann [16], and Hartmann [14], although the ego boundary in personality does not directly correspond to category boundary, it was pointed out that, when identifying a physical object, thick-boundary persons would allocate a large proportion of attention to geometric or topological property of the object rather than its featural details. In other words, they only pick out the economic, salient cue and apply it widely in perception, making room for more perceptual storage for quicker identification of other objects or finishing other cognitive tasks such as analyzing and calculating. In doing so, a person develops a cultured, rigid, and conventional mind, as Hartmann [13, 14] put it. However for the thin boundary persons, they tend to be noticing more details than necessary when identifying and categorizing objects. In Hartmann's words, this type of persons is more unstable and unconventional. This ability is very much closely associated with the recall of physical details of objects.

Cases of such were reported by Hunt and Love [17, 18] and Parker et al. [19]. The subjects can recall every factual detail such as graphic properties of printed materials but cannot understand abstract ideas. To our disappointment, from the tests these subjects were tested, only face perception and word recognition were directly related to category formation. But these subjects all reached ceiling in the tasks, indicating genius in finding detailed differences across and even within a certain category boundary. This guarantees detail and results in inefficiency, because we would be recognizing more details for categorizing a single object without the aid of a cue. A thin boundary guarantees ability to perceive within what others see as a boundary, or in other words having more dense categories. And another possibility is that their boundaries are more flexible and prone to larger changeability. Studies on speech processing favor the latter. It was found that perception of linguistic and nonlinguistic sounds is different in categorical formation. Although the perception of acoustic sound (even for linguistic sound which is nonnative) perceptual pattern is gradual; for linguistic sound it was always categorical [20]. Even thick-boundary persons may still have a fine ground gradual perception for nonlinguistic sounds. The question lies in for linguistic sound; how categorization is mapped from native language. One implication of this is to treat second language speech learning as learning nonlinguistic or purely acoustic sounds. However, that was another topic different from current inquiry.

Speech, part of the human cognitive mechanism, is usually perceived by recognizing higher-level knowledge of categories like in the process of recognizing shapes or colors [21]. One does not have to focus on all the acoustic details to comprehend a speech sound [22]. If the assumption that speech sounds as a whole is also a part of psychological categories, such as color and height, is right, which has been supported by various speech perception studies (cf. a review by [23]), then we can establish a connection between personality and language through category formation. In such studies, identification and discrimination tasks were done for sound category categorization for native languages. For example, the /p/-/b/ continuum with equal steps of voice onset time was given to native English speakers to identify and discriminate. It was shown that within-category identification rate is high and between-category identification rate is low, because the identification of sounds was constrained by the ability to pick similar tokens within the same sound category. Conversely, discrimination within a category boundary will be low because the gradient changes within a sound category was just treated as a single type of sound to our mind, which was trained in our native language (L1) learning by multiple instances of sounds and multiple speakers in a statistical way, based on the frequency of the input.

**Figure 1:**
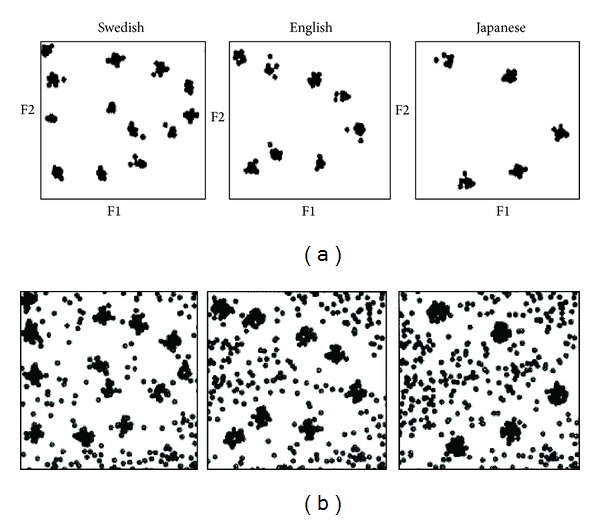
Simulations of actual adult vowel category display: instances in the F1-F2 vowel space (Kuhl and Iverson [28], upper panel), and schematic machine learning simulation results modelling an infant learning L1 or an adult learning L2 (Guenther and Gjaja [29], lower panel).

In second language (L2) speech, though the incoming speech signals are linguistic sounds, the higher-level knowledge of categories, which helps us in processing L1 automatically, might not be directly functioning because L1 and L2 categories may differ. Despite the unavailability of readily usable categories, L2 learners will “borrow” L1 categories, a process called “equivalence classification,” to increase perceptual efficiency [24] and such “laziness” of equaling L1 and L2 categories is referred to as perceptual assimilation [24, 25]. Even in cases where L1 and L2 categories are labeled as the same phoneme, L1 and L2 speakers' perception may still surface subtle mismatches [24, 26, 27]. The study of cross-linguistic acquisition of speech sounds has found that the key for correctly perceiving a nonnative speech sound is the native and nonnative sound category mapping. The formation of both L1 and L2 categories was decided by the frequency of input in the ambient language in language acquisition. Such a mapping was hypothesized to have a neurobiological basis [28]. In the process of extracting speech sounds from the ambient stimuli through speech acquisition in infancy, the extensive and continuous firing of neurons in category helps the infant establish synaptic connections between neurons within a specific area. However, for the less frequent firings, the neurons will be magnetically warped by the strong synaptic connections to ensure economic and efficient working of the cognitive device. As a result, the landscape of neural-biological firing instances is similar to the category formation as tested in the behavioral experiments. Upon the actual behavioral data from native speakers of Swedish, English, and Japanese by Kuhl and Iverson [28], Guenther and Gjaja [29] gave a neural firing simulation from a visual statistical learning programme modeling the same three languages. The results support the above hypothesis that the learning of a language can be predicted by a statistical model computed from a Bayesian algorism. See Figure 1 for the similarity of the two and replicability of the simulation. From the close resemblance of the two results, we could conclude that human cognition is very likely to be properly seen as a machine-learning computer with a normalizing function to find the statistics of, and warp up with, the incoming data into discrete categories.

## 2. Design

### 2.1. Overview

The study takes after a both qualitative and quantitative design. We have recruited only focus groups for one questionnaire-interview session and two experiments. The questionnaire-interview session finds participants' general categorical formation tendency in examining their external and internal boundaries. As expected, a positive correlation of personality and category formation is found. The first experiment was a test for participants to discriminate nonnative sounds from a language not known by the speakers. The second experiment was a test of the second language phonological categories, which the participants had learned for more than 20 years (fixed category formation).

Participants were 6 prescreened adults working as research students or administrative staff at City University of Hong Kong (3 females and 3 males, mean age = 25.83); three are identified as thick-boundary and three as thin-boundary. They are all locally raised up native Cantonese speakers who speak English as their second language. English is their working language as well. All of them are advanced speakers of English with similar IELTS score (6.5–7.5). None of them had prior exposure to other foreign languages. All participants were right-handed with no reported hearing or motor defects. They did not have prior exposure to musical training. For controlling, three native monolingual English speakers (2 female and 1 male, mean age = 26.5) from California, U.S., also participated in the study and went through the same procedure. Another 3 low-proficiency elementary English speakers (2 females and 1 male. Mean age = 25) participated in Experiment  2 as well. Each participant received HK$50 for compensation.

### 2.2. Questionnaire and Interview

The interview contains an on-site completion of personality questionnaire and a recorded confirmation talk for the researcher to decide whether the personality is depicted by the results of questionnaire. A careful screening process was done to ensure participants were of the right type. The questionnaire takes after Hartmann's Boundary Questionnaire (HBQ, [13]) to judge the type of personality. For convenience, we only used linguistically related items, as follows, and the results were sent to a frequency-based statistics analysis. The items are as follows for reference (see Table 1). The measurement is a reliable one since it has been used in a number of tests about the interrelation of participants personality and social decision-making, social orientation of various kinds, and so forth [14].

Using this measurement, the largest possible score for one participant was 200 in the current system. And adapted proportionally from Hartmann's original design, participants' score coverage of 100–200 in the continuum would be considered as relatively thick-boundary, and a score of 0–100 would be considered as thin-boundary.

A correlation between personality scores and purely unfamiliar L2 category decision (Hindi plosives, will be discussed below) was done to seek confirmation whether personality has interaction with category formation ability in human perception.

### 2.3. Experiments

The perception tests were carried out in the Phonetics Lab, City University of Hong Kong. Stimuli for both tests in both experiments were pseudowords in isolation, recorded by a native speaker of Hindi from North Delhi and an American English speaker from California, respectively. Stimuli for the first experiment were designed as perception test of Hindi velar and uvular plosive pair (/k′/-/q′/) replicated from Best et al. [30] and Best et al. [25]. Stimuli for the second experiment were designed as perception test of English minimal pairs of trVC and chVC (e.g., trep-twep). Stimuli differ in three vowel contexts, /i/, /a/, and /u/. Each word was repeated three times by the native speaker of Hindi and English, respectively, in the two experiments, and then the most clearly pronounced utterance was selected as a stimulus. Test tokens were added with the equal numbers of fillers. For the perception test, each word in the stimuli list was repeated 10 times and randomized. In total, 900 tokens were tested (6 participants × 2 experiments × 2 tasks × 3 vowels × 10 repetitions + 3 participants × 1 experiment × 2 tasks × 3 vowels × 10 repetitions).

We used these two pairs of sounds in our two experiments to investigate how unfamiliar and familiar sounds are influenced by personality. Best [24] has given an extensive prediction for how well nonnative sounds are perceived according to those sounds' mapping with the L1 sound categories. According to different perceptual distances between L1 and L2 categories, a candidate L1 sound may be perceptually identical, similar, or distinct to the target L2 sound to be perceived or learned. If the L2 sound is perceived identical phonologically to the candidate L1 sound, it will map onto the L1 category and make the L2 sound indistinguishable. For two given L2 sounds and their different perceptual distance to a given L1 sound, the PAM model proposes six possible assimilation types and predicts L2 perceiver's ability to discriminate the two L2 sounds in these situations. The types and predictions are in Table 2 as follows.

In the design of Experiment  1, we involved two speech sounds that were not probable for current participants to have contact with, for the purpose to model a non-speech-like sound environment. We expect participants to have a high discrimination rate as perceiving acoustic sounds because both sounds were not considered as speech sounds by the participants' identification, forming both uncategorizable type in PAM's prediction. The perceptual accuracy would be high. In Experiment  2, however, we designed a sound contrast of /tr/ and /t*∫*/. The latter sound was actually also produced in Cantonese as an allophonic variant for /ts/; thus it might be another candidate assimilated to /tr/ by Cantonese. Participants for pilot studies have expressed their perceptual confusion of /tr/ and /t*∫*/ [31], and thus this pair of sound is sensitive enough to mine out potential individual differences differing in personality, probably constituting a category-goodness or single-category type. The assimilation type according to the chosen sound pair in Experiment  2 conforms to the SC type, which is hard to distinguish. This makes the experiment more rigid to see different results because other easier types will not be able to unveil participants' real perceptual ability.

Both experiments involved identification and discrimination of the sounds in the minimal pairs. In the identification task, two words from a minimal pair (e.g., kik/qiq or twook/trook) were played in sequence, and the participants were asked to choose and circle either “same” or “different” on an answer sheet. In the discrimination task, three consecutive words (e.g., treek/tweek/tweek) were played, where the third word was identical to either the first or the second one. The participants were asked to circle the correct word on the answer sheet. The between-trial inter-stimulus-intervals (ISI) were set at 250 milliseconds for both tasks, and within-trial ISI at 50 milliseconds.

## 3. Results

This section reports the results of the study. It first presents the qualitative results of the interview of the participants' personality traits in a form of profile and introduces the relationship between personality type and ego boundary scores. Then, this section presents the results of behavioral tests of participants' perceptual accuracy of L2 sound contrasts. The discrimination results of Hindi stop contrast are presented first, followed by those of the English contrast. Finally, an analysis of correlative interaction between ego boundary and L2 speech perception accuracy is shown.

### 3.1. Interview

#### 3.1.1. Profiles of Participants

For a greater effect as well as efficiency due to lack of time, participants were prescreened from a pool of 20 applicants. The researcher applied intuitive observation to screen 6 most extreme cases (3 possible thick-boundary and 3 possible thin-boundary participants) as a focus group. The observation decision was based on their interest and experience in short talks before the questionnaire and interview, which are more time-consuming. Background information of the final screened participants are as follows.

1. CT is a research staff in CityU. He is 28 and is very active in learning English for academic purposes because he writes scholarly articles in English. His IELTS score is 6.5. He is a person very strict to himself and conforms to every rule and deadline.
2. DL is a Chinese Major. He is 23. He was a very hardworking student. He had an excellent GPA of 3.7. His IELTS score is 7. He often complains that he needs to learn many times before attaining good academic results because he was “often disturbed by petty details such as the font and style of a document and tone quality of a talker.”
3. KM is a part-time MA student at CityU studying translation. She is 26. She was at the same time a staff member at the Human Resources Office at CityU. Her job was very demanding in that it requires communicative and interpersonal skills in addition to English. Her IELTS score is 7.5. She is outspoken and has a very sharp eye on her surroundings and is thus a good mediator.
4. PF is a colleague of KR's. She is 28. Though some years older, she is a very keen fan of English culture. She has an IELTS score of 7. She mentioned in the interview that she has low tolerance to breaking of rules.
5. PK is an engineering student. She is 24. Her IELTS score is 7. She felt sensitive to the environment and often bombarded with “unrelated feelings and emotions.” She thought she is not suitable for engineering studies for lack of “analytic capability.”
6. SK is a very active MA student in Chinese literature. He is 26. He runs for the president of the postgraduate student union and has to prepare campaign speeches in English. He has an IELTS score of 7. However, he describes himself as an introvert and has a person with sharp eyes on events usually neglected by others.

#### 3.1.2. Results for HBQ and Interview

The result was consistent for HBQ tests and interview confirmation. Because we had already screened the participants in an impressionistic way before the actual questionnaire answering took place, it would not be surprising that the results were quite polar for thick and thin boundary participants. The interview afterwards confirmed the pattern as well, with a slight exception for KM since she displayed an introvert personality in the interview. See Table 2 for a view of the results for individual participants.

### 3.2. Results for Experiments

Results for the experiments showed that both Experiments  1 and  2 had witnessed thick-boundary and thin-boundary participants having different sound perception sensitivity. For all three comparisons, the group of participants with high ego boundary had significantly lower accuracy rate in the perception test. In Experiment  1, the rates were 60% and 81%, respectively, for thick- and thin-boundary persons [*t* = −5.435, df = 597, *P* < 0.0001], and for Experiment  2, the respective rates were 55% and 72% [*t* = 4.245, df = 597, *P* < 0.01]. The difference is significant.

However, the difference in Experiment  1 was higher than  2. Between-group difference in both Experiments  1 and  2 was statistically significant. The difference between high and low proficiency speakers' performance was significant as well. Specially, the difference in rates between thick- and thin-boundary beginning learners in Experiment  2 was 52% and 65% in Experiment  2 [*t* = 1.105, df = 297, *P* < 0.01]. The difference was significant as well.

For Experiment  1, ANOVA test results of the identification task show that the vowel effect on the accuracy rate was significant [*F*(4, 145) = 5.387, *P* < 0.001]. Discrimination test is significant as well [*F*(4, 145) = 4.396, *P* < 0.05]. Tukey's post hoc tests showed no effect of vowel [*F*(2, 498) = 0.927, *P* = 0.476]. Individual difference of the accuracy rates among three participants was not significant [*F*(2, 498) = 1.833, *P* = 0.162].

For Experiment  2, ANOVA test results of the identification task showed that the vowel effect on the accuracy rate was significant [*F*(4, 145) = 7.487, *P* < 0.001]. Discrimination test was significant as well [*F*(4, 145) = 2.768, *P* < 0.05]. Tukey's post hoc tests showed no effect of vowel [*F*(2, 498) = 0.871, *P* = 0.254]. Individual difference of the accuracy rates among three participants was not significant [*F*(2, 498) = 2.145, *P* = 0.741].

The difference between high-proficiency and low-proficiency participants was significant [*t* = 8.476, df = 670, *P* < 0.01]. The difference between the two experiments was marginally significant [*t* = 1.454, df = 967, *P* = 0.097].

**Figure 2:**
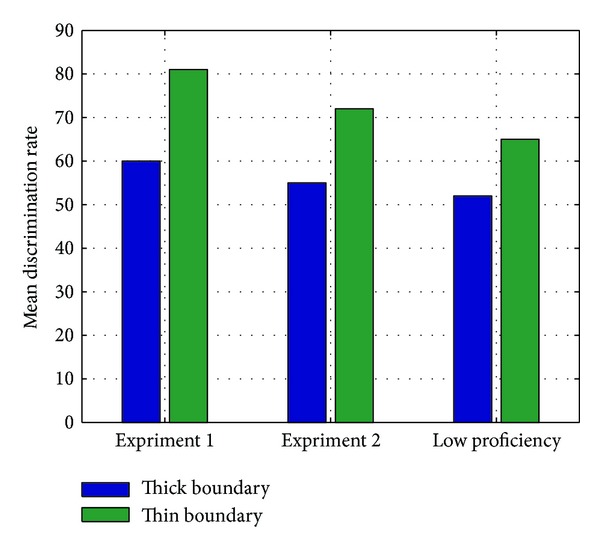
Comparison of thick-boundary speakers' (blue) and thin-boundary speakers' (green) perceptual accuracy in distinguishing purely nonnative (Experiment  1, left) and L2 (Experiment  2, middle) sound category pairs, supplemented with data from low proficiency speakers (right).

Figure 2 shows the comparison of perceptual accuracy rates of thick and thin boundary learners in Experiments  1 and  2 and low proficiency group.  Table 3 reveals the level of significance of above comparisons.

### 3.3. Interaction of Personality and Category Formation

A correlation test (Spearman) was done for the individual participants' HBQ numerical results and the average accuracy rate in both experiment rates. The correlation coefficient was significant in both Experiment  1 (*r* = 0.67, *P* < 0.05) and Experiment  2 (*r* = 0.55, *P* < 0.05). It showed that personality and category formation had a significant positive correlation.

## 4. General Discussion

The clear positive link between HBQ questionnaire results and behavioral data from perception tests was in line with our prediction that personality does have an impact on some linguistic ability, here, specifically, phonological encoding, which is closely bounded with human categorization capability.

The results correspond with Ehrman's examples [15] displaying boundary, language aptitude test scores, and others. Although the book does not discuss the boundary's relationship with any particular aspect of language acquisition outcome/performance, it is found that boundary scores are significantly correlated with the phonological encoding and phonological awareness of the students. This is consistent with our study, where the pronunciation of the participants was correlated with boundary score.

A larger difference of the two groups which occurred in Experiment  1 rather than in Experiment  2 may imply that L2 pronunciation, with a long learning period, would be influenced by other chaotic factors. Noticeably, the difference in Experiment  1 did reach statistical significance, whereas in traditional predictions of L2 category formation such as Best's [24] and Kuhl and Iverson's [28], will predict gradient perception, which is with good sensitivity to distinguish even within the category boundary. However, in the current results, the thin-boundary subjects significantly outperformed the thick-boundary ones, making the perception for gradual stimuli also differ. Despite the simplicity in the procedure in using identification and discrimination to represent perceptual sensitivity, we could draw a suggestion that personality may have an effect on the general perceptual ability of human mind, including the perception of speech-like and non-speech-like sounds. Such a finding may provide side proof for the broad idea that the nature of language being cognitive.

Another interesting finding we can draw is inspired by the low perceptual accuracy for low-proficiency speakers, but the difference was smaller. Proficiency does have an influence on the perception, and it may result in lower accuracy than a language never been encountered before, as the case in Experiment  1, partly because the Cantonese category of /ts/ is similar to both of the two English categories. This suggests that L1 influence may warp the category formation of the L2 sounds, and such warping will blur the effect of personality because L1 transfer would be a problem for both thick-boundary and thin-boundary language users.

A related concern is that thick-boundary speakers' perceptual accuracy did not see significant variation across the familiarity towards the target language. This possibly means that thick-boundary individuals would not distinguish linguistic and nonlinguistic sounds and not be interested in novel stimuli in the environment. This actually echoes with Hartmann's [13] observation that thick-boundary persons are too conventional and rely much on readily usable cues rather than analyzing themselves. Contrary to previous literature focusing on the universal distinction of linguistic and nonlinguistic sound perception, the current study actually found an individual difference on interparticipant distinction of linguistic and especially nonlinguistic perceptual capability from different personality groups. This could also crack a gap for future studies of individual difference of language learning.

The significant difference in vowel contexts, however, supports the view that linguistic sound is somewhat different from purely acoustic stimuli. For both thick-boundary and thin-boundary participants, vowel effect is significant within a phonological category. This means that phonological determined categorization is not fine-grained enough to be equalized with mental categorizations. Although this falls out of the scope of the research question of the present study, it further proves the instability of phonological representations and the necessity to analyse linguistic capacity with measurements other than language* per se*.

A profound implication of all the above observation is that cross-linguistic perception of speech sounds may be influenced discretely by psychological factors that were integral of human perceptual habits. After all personality is cultivated by complicated sets of cognitive inclinations and schema, shaped by life experience of various kinds. Practical application of this study lies in two-fold. First is predicting language learning outcome. This can be seen as a supplement to language aptitude tests. Moreover, this interconnection of three aspects can be used in classrooms. Teachers can focus on thick-boundary students and give them form-focused instruction of pronunciation in helping them to pay more attention to the details of acoustic and articulatory features of the speech sounds.

Considering the neurobiological basis of category formation, we could further infer that one's personality will influence linguistic perception by directly exerting an influence on the cognitive ability, since the difference does not differ much in types of linguistic contrast. Even for sounds usually perceived as nonlinguistic such as the Hindi plosive contrast, and for two English contrasts usually perceived as a single category, significant individual differences in personality have surfaced.

Automatic Selective Perception (ASP) model by Winifred Strange [32] proposed that individual difference in L2 speech acquisition can be seen as a function of an online process of allocating different cognitive attentional resources and formation of different selective perceptual routines. Such routines are determined by statistical learning of L1 and L2 experience and the training tasks. Here, by inheriting the results in the current study, individual differences of allocating attention may be explained by ego boundary variations.

We agree that the way we conceive the second language speech acquisition procedure should be an online cognitive computation process rather than a metaphorical construction of possible routines of perception such as equivalence classification and categorical formation. The current study's finding of personality being an effective factor of language acquisition can be incorporated into that model.

One last remark concerns how far we can draw a conclusion from this study. The current results may only exemplify the interconnections of personality, category formation, and speech at its best: it makes no attempts to argue for causality among them. Even if the results did suggest that language is not a very peculiar aspect of cognitive ability in terms of perceiving speech-like and non-speech-like sounds, we are not crowning personality as a discrete cause for such a distinction. Similar inquiry of language and thoughts had already been too controversial [33, 34]. However, it does try to provoke some contemplation into the understudied realm of interactions between behavioral science and language education.

## Figures and Tables

**Table 1:** Chosen questions in the HBQ questionnaire.

Category 3 (16 items, score range 0–64): boundaries related to thoughts, feelings, and moods, for example, merging of thinking and feeling.	
Category 4 (6 items, score range 0–24): boundaries related to childhood, adolescence, and adulthood, for example, how connected you feel to your childhood feelings.	
Category 5 (12 items, score range 0–48): interpersonal boundaries.	
Category 6 (5 items, score range 0–20): sensitivity.	
Category 7 (11 items, score range 0–44): neatness, exactness, and precision.	

**Table 2:** Results for HBQ test score and interview result by individual subjects.

Participant	Results in HBQ	Results confirmed in interview
CT	189 (very thick boundary)	Extravert, thick boundary
PF	176 (very thick boundary)	Extravert, thick boundary
KM	112 (thick boundary)	Introvert, thick boundary
PK	53 (thin boundary)	Introvert, thin boundary
DL	61 (thin boundary)	Introvert, thin boundary
SK	67 (thin boundary)	Introvert, thin boundary

**Table 3:** Results by experiment for general discrimination rate for thick and thin boundary group participants, with difference between group and difference by vowel within group.

Experiment	Difference (*t*-test) between groups	Differences by vowel context
1	*t* = 5.435** (60% versus 81%)	*F* (4, 145) = 5.387
2	*t* = 4.245** (55% versus 72%)	*F* (4, 145) = 7.487**
2-low proficiency	*t* = 1.105* (52% versus 65%)	*F* (4, 145) = 8.476*

*t*/*F*-test results with one asterisk (*) stands for significance at the *P* < 0.05 level, and two asterisks (**) stands for significance at the *P* < 0.01 level or less.
